# “Since I’m a little bit more mature”: contraception and the arc of time for women in midlife

**DOI:** 10.1186/s40695-021-00062-7

**Published:** 2021-04-09

**Authors:** Amy Alspaugh, Melody D. Reibel, Eun-Ok Im, Julie Barroso

**Affiliations:** 1 UCSF: University of California San Francisco, San Francisco, USA; 2 MUSC: Medical University of South Carolina, Charleston, USA; 3 Emory University, Atlanta, USA; 4 Vanderbilt University, Nashville, USA

**Keywords:** Contraception, Middle aged, Reproductive health, Menopause, Qualitative research

## Abstract

**Background:**

Contraceptive methods have rapidly evolved over the past several decades, but little research has explored how women interact with contraception over time. Exploring contraceptive beliefs, perceptions, and attitudes of women in midlife can reveal much about how lived experience affects contraceptive decisions and reproductive health choices.

**Methods:**

Individual, semi-structured interviews were conducted with 20 women between the ages of 40 and 55 who had not reached menopause and did not have a permanent method of sterilization. Data were coded using qualitative descriptive methods.

**Results:**

Three major themes were identified: 1) journey toward empowerment; 2) finding the right fit: evolution over time; and 3) anticipating a transition. Past experiences with or fear of side effects and hormones were common reasons to change or avoid certain contraceptive methods. Most participants were happy with their contraceptive method; however, those who were unhappy were more likely to vocalize fatigue at continuing to need contraception as menopause approached.

**Conclusion:**

Approaching contraceptive counseling from a place that considers the journey with contraception over a reproductive life span will help identify how beliefs, perceptions, and attitudes of women affect their contraceptive practices and choices.

## Background

The field of contraception has undergone a remarkable evolution in the past several decades, so remarkable, in fact, that the United States’ Centers for Disease Control and Prevention named family planning one of the success stories of public health in the twentieth century [1]. Around the globe, women have seen their options and access to highly-effective contraception increase, albeit unevenly across nations with vast discrepancies in resources.

Women nearing menopause have seen this evolution take place throughout their reproductive years. A woman currently in her mid-fifties likely started her journey with contraception in the early-to-mid 1980s, when only a few types of contraception were available. A plethora of delivery systems and hormonal formulations have been introduced during the past several decades. Whether women are trying newer methods, or whether their contraceptive choices are largely unaffected by contraceptive technology advances is not well understood. Similarly, information on how past experiences, both positive and negative, influence current contraceptive decision-making is limited. In the United States (US), the most recent data collected from 2006 to 2010 indicate that women aged 15–44 use an average of 3.1 contraceptive methods during their lives [2]. These data suggest that many US women will only use a small number of the various family planning methods available.

A literature review revealed a few previous US studies that evaluated women’s perceptions and experiences with contraception over time. Two studies qualitatively explored perceptions of young women who identified as Black and Latina. Gomez et al. found previous experiences with side effects, high levels of distrust in medical providers, and the experiences of others undergirding their decision not to use a long-acting reversible contraceptive [3]. Downey et al. identified an iterative, relational, and reflective process in which young women made contraceptive decisions [4]. Another study explored the contraceptive journey of women age 16–50 quantitatively, evaluating methods used, reasons for changing a method, and interactions with healthcare providers [5]. To our knowledge, the current study is the first to address the contraceptive journey from the perspective of women in midlife, between the ages of 40 to 55, who arguably have the longest time frame of experience but are rarely included in contraceptive research [6]. Consequently, little is known about the reproductive journeys of women in midlife, who have potentially been using contraception for three or more decades [7, 8].

There is currently much debate among family planning practitioners and researchers regarding how to make contraceptive methods and counseling more patient-centric [9, 10]. Efforts to move past tiered contraceptive counseling, based on method effectiveness alone, is gaining favor as reproductive justice becomes the default framework [11, 12]. Included in this effort is consideration of the lived, personal experience of the individual as equally important, if not superior to, the evidence-based, scientific knowledge since several evidence-based, effective options are now available [4, 13]. Part of the process that is necessary to elucidate these perspectives involves feminist, womanist, and intersectional qualitative research [14]. To this end, the present qualitative analysis explores the following questions: How do women in midlife view their contraceptive journey? What shapes their current beliefs, perceptions, and attitudes about contraception? What can be learned from women in midlife that may help women across the reproductive lifespan?

## Methods

### Sample

Participants were recruited throughout the Research Triangle area of North Carolina, a mid-sized urban community comprised of several smaller cities. Information about the study and pertinent contact information was included on flyers placed at women’s health clinics and public venues that reach a wide variety of women such as libraries, grocery stores, and coffee shops. Recruitment also occurred through snowball sampling: study participants were encouraged to share information on the study with friends or family who might be interested [15]. There was no a priori number of participants we were seeking to recruit; we followed qualitative traditions by recruiting until we reached data saturation and were not able to identify any new themes or concepts [16].

The following criteria guided participant recruitment: identifying as a woman, being between the ages of 40 and 55, speaking English, and having not yet reached menopause. Exclusion criteria included having a permanent method of sterilization or having a partner with a permanent method of sterilization. Sterilizations were excluded to ensure the research focused on those still making contraceptive decisions over 40. Approval was sought from the Medical University of South Carolina’s Institution Review Board (IRB), and the study, #Pro00089636, was deemed exempt from Human Subjects Research Regulations.

### Data collection

Interested participants telephoned the first author for prescreening eligibility or filled out a secure online survey through REDCap. Those who screened eligible were contacted to set up an interview time and location. Interviews were face-to-face, individual, semi-structured, and in-depth. An interview guide steered the discussion and covered topics such as contraception, pregnancy planning, and the approach of menopause. The guide was minimally revised during data collection based on early interviews.

The interviews occurred between September and December of 2019 and were all conducted by the first author. Interviews took place at locations selected by the participants to ensure ease of access and optimal comfort level. Most often, interviews occurred at coffee shops, fast food restaurants, and public libraries. Each participant was interviewed a single time. Interviews lasted between 35 and 55 min and were audio-recorded with a handheld device and then transcribed verbatim. Participant verbal consent was obtained by the interviewer at the time of the interview. Participants were given $25 gift cards to compensate for their time.

### Data analysis

The study was conducted within a qualitative descriptive approach. The goal of qualitative description is to describe an individual’s experience in that individual’s own words at a manifest level [17]. Qualitative descriptive methodology is based on the overarching principles of naturalistic inquiry, meaning that people and their experiences are observed and interpreted within the social and cultural context of their lives [18]. First and second level qualitative coding was initiated by the first author consistent with qualitative descriptive methodology [19]. Three overarching themes were identified based on thorough review of the first and second level codes and all second level codes were organized under the relevant theme.

The fourth author reviewed all first level coding for the first 10 interviews and second level coding for every other interview using a detailed data audit trail. Additionally, the second and third authors reviewed all coding on a quarter of the interviews and provided input to the final coding matrix and themes. Areas of disagreement were discussed, and consensus was obtained among all authors. Interviews were coded in Microsoft Word and demographic data were analyzed in Microsoft Excel. Trustworthiness was enhanced through 1) prolonged engagement with the transcripts, 2) creation of an audit trail, 3) use of reflexive journaling by the interviewer, and 4) member checking with 9 participants. Member checking both assisted in confirming themes and also provided further clarification about the researchers’ interpretation and understanding of the participants responses.

## Results

Of the 32 women who completed prescreening eligibility, 22 participants met all the inclusion criteria. Of these 22 women, 20 completed the study and 2 were unable to be scheduled for interviews due to scheduling conflicts or being lost to follow-up. Participant recruitment continued until data saturation was reached and no new themes were identified. Table 1 shows the participants’ demographic information and Table 2 provides information regarding reported sexual activity in the prior 2 months.

**Table 1:** Demographic characteristic of 20 interview participants

	Frequency	Percentage
**Age, y**		
Range	40–55	
Mean	45.05	
Median	45	
**Race**^a^		
White	12	60
Black or African American	8	40
Other	1	5
**Ethnicity**		
Hispanic/Latinx	0	0
Non-Hispanic/Latinx	20	100
**Sexual Orientation**		
Heterosexual or straight	18	90
Gay or lesbian	1	5
Bisexual	1	5
**Household Income**		
Less than $10,000	3	15
$10,001 – $24,999	4	20
$25,000 – $34,999	2	10
$35,000 – $49,999	1	5
$50,000 – $74,999	1	5
$75,000 – $99,999	3	15
More than or equal to $100,000	6	30
**Marital status**		
Single	10	50
Married	8	40
Divorced or separated	1	5
Prefer not to say	1	5
**Education**		
High school diploma or GED	1	5
Some college, no degree	4	20
Trade, technical, or vocational training	1	5
Bachelor’s degree	2	10
Master’s degree or higher	12	60
**Religion**		
Catholic	2	10
Protestant	1	5
Non-denominational Christian	7	35
Jewish	1	5
Agnostic	1	5
Other	5	25
None	3	15

^a^Participants able to select more than one option

**Table 2:** Reported sexual activity of 20 interview participants

	Frequency	Percentage
**Do you consider yourself sexually active?**		
Yes	15	75
No	5	25
**Have you been sexually active in the past two months?**		
Yes	15	75
No	5	25
**Currently, with whom are you sexually active?**		
Men	18	90
Women	2	10
Both	0	0
**Currently, how many people are you sexually active with?**		
0	5	25
1	13	65
2	1	5
More than 2	1	5

Three major themes were identified: journey toward empowerment; finding the right fit: evolution over time; and anticipating a transition. Subthemes are presented below in order of most to least prevalent.

### Journey toward empowerment

Many women spoke of their contraceptive behavior at midlife in comparison to their younger selves. They were able to identify a transition in their own behavior. This manifested in becoming more confident in speaking up about the desire to use condoms, and in the ways in which they used experiences they had had with their own mothers to inform how they talked about sex and health with their own children in the present day.

#### More confidence in addressing contraceptive needs

Over half of the women interviewed reported a difficult reproductive health scenario when they were young and newly sexually active. During their first few years of sexual activity, women in this study reported adolescent pregnancy, abortion, and risky sexual behavior. Some women also reported difficulty obtaining the contraceptive method of their choice, due to family members preventing access or because of problems with insurance.

Many women in midlife described an increase in their comfort level when discussing their contraceptive needs with their partner or partners compared to when they were younger. For some women, this meant not using a method with which they felt uncomfortable. For others, it meant sharing the task of contraception with the partner, such as using male condoms, instead of feeling the need to take full responsibility for decisions and the action of using contraception all by themselves.

Specifically, several women mentioned that they felt much more confident insisting on condom use by their partners now than when they were younger. One woman discussed how she ensured safety against sexually transmitted infections (STIs): “Condoms and plus, now since I’m a little bit more mature, you’re gonna go take a test for whatever disease before I even think about having sex with you.” Women who specifically mentioned that condoms were currently non-negotiable with new partners were often the same women who had talked about their own sexual risk-taking when not using protection in their youth.

#### The role of mothers

Mothers, or mother-figures, played a large role in how women talked about reproductive and sexual health in their youth. More commonly, women reported difficult relationships with their mothers. This difficulty manifested in numerous ways, from mothers who refused to talk about sexual activity or contraception with their adolescent children to mothers who simply tossed contraceptive pills across the dinner table at their daughters without further discussion. One participant recalled how distressing it was when her mother scheduled her first well woman exam at an abortion clinic. Several women reported approaching their mothers for help in obtaining contraception only to find that help denied. One woman discussed a conversation that did not feel age-appropriate to her: “Well, when I got my period, I was 12, she told me I could get pregnant. And, um, I was still playing with Barbie dolls and, um, sex was not something that was on my mind. For a very long time, even after that conversation. So I was very confused. So she’s not a person that I would really go to about anything sexual whatsoever.”

This initial encounter had longer-term impacts that extended beyond adolescent sexual and reproductive health. As noted in the quote above, the participant felt that she could not turn to her mother with these kinds of personal questions going forward. Many women reported that they continued to feel strained in health-related conversations with their mothers. Thus, women found themselves without full knowledge of their family health history or information about their mother’s experience with menopause.

However, women who had adolescent or young adult children prioritized talking to their children about contraception and in many cases helped them select or procure their desired method. Women who had negative experiences with their own mothers intentionally tried to create open and accessible dialogue with their own children. They expressed a desire for the kind of communication and respect that they, themselves, did not receive when they were the age of their children. One participant noted: “Basically a lot of things that my mom did that I didn’t like, I made sure that I didn’t do them with my child. Things like... I kind of used the reverse, um, kind of like, this is what not to do as a parent.” Reclaiming the role of helper and advisor for their children was empowering and essential for these women.

### Finding the right fit: evolution over time

Many women reported using fewer than 4 methods during their lifetime. Notably, half of the women in this study had not used a hormonal method other than the pill. Many of the women who had continued with the same method for decades reported no problems with side effects and general ease of use that kept them from needing to change methods. A smaller number of women currently using a barrier or non-hormonal method reported previous side effects of hormonal contraception that made them wary of all hormonal methods.

Among women who had used more than 4 methods during their life, many discussed changing over the years to find a method that better aligned with their lifestyle. For several women, this meant gradually shifting toward methods with smaller doses of hormones. For others, it meant exploring nondaily dosing options or switching to a coitus-dependent method such as condoms, if they were not frequently sexually active.

#### Hesitancy with hormonal contraception

Concern regarding the use of hormonal contraception was frequently discussed by participants. Some reported their hesitancy regarding hormones had existed since they started using contraception in their youth. Avoiding pregnancy was so important to these women when they were younger that they ignored their own hesitancy. As they got older, however, they felt empowered to select a method that was more congruent with their desire to avoid exogenous hormones. One woman said, “I’ve always been leery of [hormones], I just really wanted to protect myself from becoming pregnant.” In a few instances, use of non-hormonal methods was made easier by an increase in their knowledge about their own reproductive physiology, which helped them feel more confident using fertility awareness or a barrier method. So, while beliefs regarding hormones did not change, the ability to act on them did.

Other women vocalized a more recent hesitancy to use hormonal methods. For some women, this meant not using hormonal methods at all, but for others, it meant using methods with a lower or more localized dose. Many women expressed a desire to have more bodily awareness or a natural hormonal balance and were looking for methods that allowed for hormonal fluctuations, ovulation, or both. One woman who had used contraceptive pills for many years expressed concern about the length of time she had been using hormonal contraception:

“Um, when I was younger I used the pill, I was not worried about hormones when I was younger. Like, I knew there was (sic) risks to it, but when I was young I was like “eh, whatever, this is the easiest and the best way to do it, and I’m just gonna do it.“ And then as I’ve gotten older I’ve thought “You know what? I don’t think I should really be on the pill nonstop for twenty, thirty years.“ So, the thought of like, what something is doing to my body has gotten more pronounced as I’ve gotten older. I care more about what it’s doing to me.”

#### Experiences with side effects or perceived risks

Although some women, especially those who had been using the same methods for several decades, reported no problems with side effects, many women had experienced side effects that influenced their perceptions of the safety and ease of use of contraception. For two women, a family history of breast cancer affected their perceptions regarding the safety of hormonal contraception, especially regarding a method containing estrogen and at their current ages. Others had experienced a decrease in their sex drive, an exacerbation of fibroids, and/or fatigue that they attributed to their hormonal contraception since the fatigue improved when they stopped using that particular method. A small number of women said that side effects they experienced, even if those side effects happened several decades in the past, would likely deter them from using a hormonal method again.

#### Deciding to change methods

While the majority of women made these decisions on their own, a few women specifically mentioned the role of their health care provider in prompting them to explore another option. Generally, these suggestions were met positively, as long as the women felt the guidance was individualized to their specific situation in life. Suggestions from the health care provider to change a method in the absence of an identified problem, however, were met with suspicion and distrust by women in this study.

#### Contraceptive dissatisfaction and fatigue

A small number of women reported dissatisfaction with their current contraceptive method; their current method felt like a stopgap measure that would see them through to menopause. Women who were unhappy with their current method of contraception were more likely than women who were satisfied with their method to report feeling fatigued with continuing to deal with contraception in midlife. One woman expressed the following: *“*Me and birth control are ready to break up … I’d say it was a newness in my 20s, and a steady relationship, you know, in my 30s, but it’s kind of time to have that conversation to end things.” Interestingly, all of the women who reported dissatisfaction with their contraceptive method felt that it was very important to use a method until reaching menopause.

### Anticipating a transition

Women in midlife are certainly aware of the existence of menopause and its present or future impact on their lives. To women in this study who did and did not desire more children, midlife was a time of shifting health priorities. One participant explained the transition by saying: “I think you get, you get away from like let’s make sure your body’s ready for a baby to let’s make sure you don’t die at 50.”

#### Acknowledgement of health needs for a changing body

Almost all participants spoke of prioritizing their own personal health and wellness. While most did not see menopause as a big, life-changing event, it did make them start to think about how they should enter older adulthood by prioritizing themselves. Women spoke of trying to eat better, exercise more, and integrate exercise that would counteract the stiffness and decreased pliancy they already felt in their bodies. This view toward long-term health was not new for all women, but it was something most of them shared in common. One woman spoke of her own shift toward considering her health in midlife: “Yeah, you know what, this is the time to start you know, prevent it- you know go ahead and make you’re sure- checking on myself, now that I’m getting older.” While reproductive health was important, a shift in priorities seemed to be occurring for women in midlife. Their own personal health and wellness could no longer be put on the backburner. One participant summed it up saying: “It’s not about sex and having babies. It’s about, like, staying healthy.”

#### Continuing to explore the possibility of pregnancy

While most of the women reported no plans for pregnancy in midlife, seven of the twenty participants were actively trying to get pregnant or seriously considering a pregnancy in the future. Women actively trying to get pregnant expressed some concern about the likelihood of pregnancy given their age but felt hopeful and excited at the prospect of a becoming a mother in midlife. The women considering another pregnancy all reported valuing a method that gave them the ability to stop a method quickly and easily and allowed for a quick return to fertility. Speaking generally of women having children at older ages, one participant said: “I think that’s a trend that’s not going to change particularly in terms of pregnancy and having children, um, and so I think there is sort of room to know more and kind of adjust our services and our attitudes accordingly to think about how we can adjust the needs of women in their late 30s, early 40s with having kids, and kind of meet them where they are...”

#### Interest in other methods

Although most of the women interviewed were satisfied with their contraceptive method, some of these women vocalized interest in other methods. Primarily, women discussed that they would use an intrauterine device (IUD), especially the levonorgestrel system, if they were younger. Some had used the method before and liked it but felt that there would not be enough time left before menopause to use it to justify the hassle of getting an appointment to have it placed.

## Discussion

The aim of this study was to explore the ways in which the arc of time, including the events of the past as well as looking toward the future, influenced the contraceptive beliefs, preferences, and attitudes of women in midlife. From reviewing extant literature, it appears that this research is among the first to qualitatively address the contraceptive journeys of midlife women. Women in midlife, like women across the lifespan, make contraceptive decisions based on numerous individual factors. Pregnancy prevention and method effectiveness, once gold standards in family planning research and development, may or may not be strongly considered at all life stages by all individuals [13, 20]. For women in this study, contraception in midlife was something to be considered within the greater context of their lives, instead of as their primary focus as it may have been when they were younger.

First experiences with seeking contraception are important life events that are often remembered long after they occur. Parents and guardians are often the gate-keepers of information on sexual health and access to contraception. However, for many participants in this study, parents were a barrier to evidence-based information. To reduce these barriers, there needs to be a concerted effort to ensure that parents and guardians have the right information and training to know how and when to talk to their children about sex and contraception. Evidence-based educational efforts are necessary to ensure parents and guardians have the tools to fulfill this important function [21, 22]. At a clinical and policy level, it is essential to ensure that women have access to affordable or no-cost contraception at any age. Over-the-counter contraception, ensuring parent consent is not required for any family planning service including abortion, and more targeted education in schools can help address this at the policy level [23–25]. Clinically, providers should ensure individual time with adolescent patients to discuss topics like contraception is part of every appointment [26].

Additionally, women who reported negative experiences with their mothers during adolescence were often the same women who did not, in later years, feel comfortable asking about menopause or their family’s health histories. This reluctance or inability to explore one’s family medical history could result in missed opportunities for screenings and other types of intervention.

Women in the present study expressed hesitation at the use of exogenous hormones for contraception. Women were split between those who had felt this way their entire adult life and those who had become more wary with age, because of an experience with side effects or a concern over specific medical conditions. These findings mirror the results of a small number of US-based studies focused on adolescent and young women, all of which pointed to a hesitancy regarding hormones that is not often discussed clinically [27, 28]. Contradictory results about the safety and risks of hormone therapy from the Women’s Health Initiative study further compound this hesitancy, especially for women in midlife [29]. Even those women without specific fears regarding hormones spoke to concerns regarding side effects of contraception. These findings may indicate a need for further patient education about the safety of hormonal contraception in midlife. Alternatively, it may point to a need for more targeted research on the effects of the exogenous hormones used in contraception specifically in women in midlife, as this age-group is often left out of contraceptive research.

Also clinically relevant was the finding that many women vocalized interest in a different method but felt that it was too late to initiate a new method because of the amount of time remaining before menopause. Many of the women who expressed this concern were specifically referring to the levonorgestrel-releasing IUD, which, at least theoretically, may decrease potentially bothersome perimenopausal symptoms such as irregular bleeding [30, 31]. Women reach menopause on average at the age of 51, meaning that many of the women who vocalized interest in an IUD would likely have experienced many years of highly effective pregnancy prevention and perhaps even a reduction in perimenopausal symptomology [32]. Because hormonal contraception often masks the symptoms of perimenopause, it is often difficult to identify the onset of menopause in individuals using hormonal contraception. Thus, for those individuals who wish to continue using hormonal contraception, clinicians should specifically relay information from clinical guidelines [32] that encourage continuation of contraception until age 55. Knowledge of the specifics of this time frame may assist women in creating the best contraceptive plan for their needs and desires.

This study was limited in the following ways: Despite the overall high percentage of participants who were a racial or ethnic minority, had a lower socioeconomic status, or were a sexual minority, many racial and ethnic groups were not represented in this research. Specifically, because speaking English was part of the inclusion criteria, non-English speakers, a significant population in this geographic area, were not able to participate. Additionally, this study does not include the experiences of those women who selected a surgical method of contraception. Because contraception is used not only to prevent pregnancies but also to address a host of gynecologic concerns, focus on these communities and other populations, including sexual and gender minorities, would be beneficial. Additionally, the positionality of the first author who conducted the interviews, as a white, cisgendered, heterosexual female who worked as a women’s health care provider, may have exacerbated imbalances of power, privilege, and knowledge creation among study participants [33].

## Conclusions

Women in midlife are a dynamic and empowered group. Many still desire pregnancy while others have moved on to another stage of their lives. Some adore their combined hormonal pills and others have long ago written off exogenous hormones, but they are similar in their consideration of long-term planning regarding their personal health. If the past two decades are any indication, contraception will continue to evolve to include new devices, delivery systems, and hormonal formulations. By listening to women in midlife who have been privy to contraceptive changes during their lifetimes, we can gain greater understanding of contraceptive beliefs, priorities, and attitudes. The ability to access, use, and control contraception when it is desired is a hallmark of reproductive justice and personal autonomy across the lifespan [13].

## Abbreviations

IUD: intrauterine device
STI: sexually transmitted infection

## References

[1] Ten great public health achievements--United States (1999). 1900-1999. MMWR Morb Mortal Wkly Rep.

[2] Daniels K, Mosher W. Contraceptive methods women have ever used: United States, 1982-2010. Natl Health Stat Rep. 2013;(62):1–15. 24988816

[3] Gomez AM, Arteaga S, Aronson N, Goodkind M, Houston L, West E (2020). No perfect method: exploring how past contraceptive methods influence current attitudes toward intrauterine devices. Arch Sex Behav.

[4] Downey MM, Arteaga S, Villasenor E, Gomez AM (2017). More than a destination: contraceptive decision making as a journey. Womens Health Issues.

[5] Nelson AL, Cohen S, Galitsky A, Hathaway M, Kappus D, Kerolous M, Patel K, Dominguez L (2018). Women's perceptions and treatment patterns related to contraception: results of a survey of US women. Contraception..

[6] Woods NF, Bachmann G (2017). Contraception for midlife women: lack of information? Lack of interest? Lack of investment?. Womens Midlife Health.

[7] Harlow SD, Dusendang JR, Hood MM, Woods NF (2017). Contraceptive preferences and unmet need for contraception in midlife women: where are the data?. Womens Midlife Health.

[8] Alspaugh A, Barroso J, Reibel M, Phillips S (2020). Women’s contraceptive perceptions, beliefs, and attitudes: an integrative review of qualitative research. J Midwifery Womens Health.

[9] Foster DG (2016). Unmet need for abortion and woman-centered contraceptive care. Lancet.

[10] Dehlendorf C, Reed R, Fox E, Seidman D, Hall C, Steinauer J (2018). Ensuring our research reflects our values: the role of family planning research in advancing reproductive autonomy. Contraception..

[11] Brandi K, Fuentes L. The history of tiered-effectiveness contraceptive counseling and its effects on patient-centered family planning care. Am J Obstet Gynecol. 2019;222(4S):S873–S877. 10.1016/j.ajog.2019.11.1271. 10.1016/j.ajog.2019.11.1271 31794724

[12] Higgins JA (2014). Celebration meets caution: LARC’s boons, potential busts, and the benefits of a reproductive justice approach. Contraception..

[13] Potter JE, Stevenson AJ, Coleman-Minahan K, Hopkins K, White K, Baum SE, Grossman D (2019). Challenging unintended pregnancy as an indicator of reproductive autonomy. Contraception..

[14] Im EO (2013). Practical guidelines for feminist research in nursing. Adv Nurs Sci.

[15] Moser A, Korstjens I (2018). Series: practical guidance to qualitative research. Part 3: sampling, data collection and analysis. Eur J Gen Pract.

[16] Sandelowski M (1995). Sample size in qualitative research. Res Nurs Health.

[17] Willis DG, Sullivan-Bolyai S, Knafl K, Cohen MZ (2016). Distinguishing features and similarities between descriptive phenomenological and qualitative description research. West J Nurs Res.

[18] Colorafi KJ, Evans B (2016). Qualitative descriptive methods in health science research. Herd..

[19] Sandelowski M. Whatever happened to qualitative description? Res Nurs Health. 2000;23(4):334–40. 10.1002/1098-240X(200008)23:4<334::AID-NUR9>3.0.CO;2-G. 10.1002/1098-240x(200008)23:4<334::aid-nur9>3.0.co;2-g 10940958

[20] Callegari LS, Aiken AR, Dehlendorf C, Cason P, Borrero S (2017). Addressing potential pitfalls of reproductive life planning with patient-centered counseling. Am J Obstet Gynecol.

[21] Santa Maria D, Markham C, Bluethmann S, Mullen PD (2015). Parent-based adolescent sexual health interventions and effect on communication outcomes: a systematic review and meta-analyses. Perspect Sex Reprod Health.

[22] Ford CA, Mirman JH, Garcia-Espana JF, Fisher Thiel MC, Friedrich E, Salek EC (2019). Effect of primary care parent-targeted interventions on parent-adolescent communication about sexual behavior and alcohol use: a randomized clinical trial. JAMA Netw Open.

[23] Fuentes L, Ingerick M, Jones R, Lindberg L (2018). Adolescents’ and young adults’ reports of barriers to confidential health care and receipt of contraceptive services. J Adolesc.

[24] Lopez LM, Bernholc A, Chen M, Tolley EE. School-based interventions for improving contraceptive use in adolescents. Cochrane Database Syst Rev. 2016;(6):Cd012249. 10.1002/14651858.CD012249 PMC9239532 27353385

[25] Wilkinson TA, Miller C, Rafie S, Landau SC, Rafie S (2018). Older teen attitudes toward birth control access in pharmacies: a qualitative study. Contraception..

[26] Santa Maria D, Guilamo-Ramos V, Jemmott LS, Derouin A, Villarruel A (2017). Nurses on the front lines: improving adolescent sexual and reproductive health across health care settings. Am J Nurs.

[27] Coombe J, Harris ML, Loxton D (2019). Motivators of contraceptive method change and implications for long-acting reversible contraception (non-)use: a qualitative free-text analysis. Sex Reprod Healthc.

[28] Chernick LS, Schnall R, Higgins T, Stockwell MS, Castano PM, Santelli J (2015). Barriers to and enablers of contraceptive use among adolescent females and their interest in an emergency department based intervention. Contraception..

[29] Langer RD (2017). The evidence base for HRT: what can we believe?. Climacteric..

[30] Wildemeersch D (2016). Why perimenopausal women should consider to use a levonorgestrel intrauterine system. Gynecol Endocrinol.

[31] Miller TA, Allen RH, Kaunitz AM, Cwiak CA (2018). Contraception for midlife women: a review. Menopause.

[32] ACOG Practice Bulletin No. 141: management of menopausal symptoms. Obstet Gynecol. 2014;123(1):202–16. 10.1097/01.AOG.0000441353.20693.78 24463691

[33] Muhammad M, Wallerstein N, Sussman AL, Avila M, Belone L, Duran B (2014). Reflections on researcher identity and power: the impact of positionality on community based participatory research (CBPR) processes and outcomes. Crit Sociol.

